# RANKL-RANK-OPG Pathway in Charcot Diabetic Foot: Pathophysiology and Clinical-Therapeutic Implications

**DOI:** 10.3390/ijms24033014

**Published:** 2023-02-03

**Authors:** Tommaso Greco, Antonio Mascio, Chiara Comisi, Chiara Polichetti, Silvio Caravelli, Massimiliano Mosca, Nicola Mondanelli, Elisa Troiano, Giulio Maccauro, Carlo Perisano

**Affiliations:** 1 Orthopedics and Trauma Surgery Unit, Department of Ageing, Neurosciences, Head-Neck and Orthopedics Sciences, Università Cattolica del Sacro Cuore, Fondazione Policlinico Universitario A. Gemelli IRCCS, 00168 Rome, Italy; 2 U.O.C. II Clinic of Orthopaedics and Traumatology, IRCCS Istituto Ortopedico Rizzoli, 40136 Bologna, Italy; 3 Department of Medicine Surgery and Neurosciences, University of Siena, 53100 Siena, Italy

**Keywords:** charcot foot, RANKL, OPG, osteoarthopathy, diabetes mellitus, denosumab, bisphosphonates, calcitonin

## Abstract

Charcot Foot (CF), part of a broader condition known as Charcot Neuro-Osteoarthropathy (CNO), is characterized by neuropathic arthropathy with a progressive alteration of the foot. CNO is one of the most devastating complications in patients with diabetes mellitus and peripheral neuropathy but can also be caused by neurological or infectious diseases. The pathogenesis is multifactorial; many studies have demonstrated the central role of inflammation and the Receptor Activator of NF-κB ligand (RANKL)-Receptor Activator of NF-κB (RANK)-Osteoprotegerin (OPG) pathway in the acute phase of the disease, resulting in the serum overexpression of RANKL. This overexpression and activation of this signal lead to increased osteoclast activity and osteolysis, which is a prelude to bone destruction. The aim of this narrative review is to analyze this signaling pathway in bone remodeling, and in CF in particular, to highlight its clinical aspects and possible therapeutic implications of targeting drugs at different levels of the pathway. Drugs that act at different levels in this pathway are anti-RANKL monoclonal antibodies (Denosumab), bisphosphonates (BP), and calcitonin. The literature review showed encouraging data on treatment with Denosumab, although in a few studies and in small sample sizes. In contrast, BPs have been re-evaluated in recent years in relation to the high possibility of side effects, while calcitonin has shown little efficacy on CNO.

## 1. Introduction

Charcot Foot (CF), part of a broader condition known as Charcot Neuro-Osteoarthropathy (CNO), is a chronic and degenerative disease characterized by a progressive loss of protective sensibility in the foot and ankle which, through repeated trauma, leads to destruction of bone, joints, and surrounding structures [1]. It is one of the most devastating complications of patients with diabetes and peripheral neuropathy [2].

CNO is related to several neurological, infectious diseases and toxic syndromes such as alcohol abuse [3,4]. In 1868, for the first time, Jean-Martin Charcot described the CF as a complication in patients with tabe dorsalis (myelopathy due to syphilis), whereas in 1936 William Reilly Jordan described foot and ankle CNO as a complication of diabetes for the first time [2]. The joints most affected by CNO are the tarsal, metatarsals, and phalanges, although localizations such as the knee, hip, wrist and spine have also occurred [5,6,7,8].

There are two main classification models of CF: the Modified Eichenholtz classification [9], which relies on clinical and radiographic findings, and the Brodsky classification [10], which focuses on the anatomical distribution of the affected bone segments. The modified Eichenholtz classification ranges from Stage 0 (acute-inflammatory phase), in which the patient presents only cutaneous signs but no visible changes on radiography, to Stage 1 (fragmentation phase), with radiographic evidence of bone destruction, dislocation or subluxation, to Stage 2 (coalescence phase), with fusion of large fragments of adjacent bones and new periosteal bone formation, up to Stage 3 (consolidation phase) with remodeling and new bone formation with possible gross residual deformity [9]. According to Brodsky’s classification, CNO of the foot initially occurs in the Lisfranc joint (tarsometatarsal) and in the tarsus minor (type 1); type 2 occurs in the transverse tarsus (Chopart’s joint), subtalar and peritalar. Type 3a involves the ankle and tibio-talar joints, while type 3b also involves the calcaneus with associated Achilles tendon insufficiency [10].

In the acute phase of CF, generally in afebrile patients with normal vital signs and infection blood markers, the foot is characterized by swelling, warmth and erythema. Usually, skin temperature is increased (from 2 °C to 8 °C) compared to the contralateral; pain may not always be present due to diabetic peripheral neuropathy (this occurs approximately in 50% of cases) and can be associated with impaired deep tendon reflexes, especially the Achilles reflex [11,12].

This phase, often undiagnosed and rapidly progressive, leads to the chronic phase of the disease, with severe deformity, prominences of the bone and a structurally deformed foot [13]. A late and pathognomonic sign is a “rocker bottom foot”, characterized by a prominent calcaneus/heel and a convexly rounded sole [14]. Diagnosis is based on the clinic, often requires radiography, computed tomography (CT) and magnetic resonance imaging (MRI) for any differential diagnosis, especially in the acute and fragmentation phase with bone destruction [15,16,17].

Surgical treatment is reserved for the treatment of deformities and ulcers in the chronic phase; the type of deformity and the patient’s condition will lead to different types of surgical treatment, such as exostectomy, arthrodesis (by internal or external fixation) or amputation [18].

In recent decades, CF is described as a disease with an increased inflammatory response and osteolysis [19], hence many authors have focused on inflammatory and bone remodeling pathways in CNO to better understand their pathogenetic mechanisms and possible therapeutic implications [13,20,21].

The aim of this narrative review is to analyze the Receptor Activator of NF-κB ligand (RANKL)-Receptor Activator of NF-κB (RANK)-Osteoprotegerin (OPG) pathway in CF, highlighting its role in bone remodeling and its correlation with the inflammatory response, and to evaluate the possible therapeutic implications of targeting drugs at different levels of the pathway based on the available literature.

## 2. RANKL-RANK-OPG Signaling Pathway in Bone Remodeling

Bone remodeling is orchestrated by the activity of osteoblasts (OBs), which produce new bone, and osteoclasts (OCs), that instead reabsorb it. OBs are mononuclear cells derived from mesenchymal stem cell precursors (MSC) [22], and OCs are large multinucleated cells derived from a hematopoietic precursor via the fusion of progenitor cells of mononuclear osteoclasts (OCs) [23].

In 1981, Rodan and Martin hypothesized that OCs genesis was regulated by OBs [24]; while in the mid-1990s it was discovered that this regulation occurs through the expression of members of the Tumor Necrosis Factor (TNF) superfamily, such as the Receptor Activator of NF-κB ligand (RANKL) and Osteoprotegerin (OPG), with the activation of Receptor Activator of NF-κB (RANK) on the cell membrane of the OCs precursors [25].

**Figure 1:**
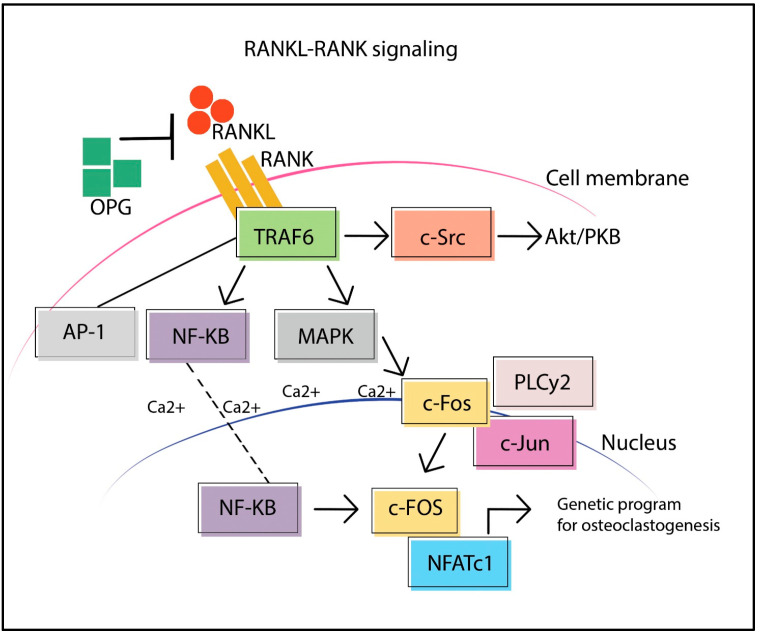
RANKL-RANK-OPG signaling pathway.

RANKL-RANK signaling is essential for OCs genesis and differentiation. RANKL, a transmembrane protein resident in the cell membrane of OBs and their precursors, can be released from the membrane by proteolysis from extracellular proteases (disintegrin and/or metalloprotease-7) [26], by activating the RANK receptor located on the cell membrane of the OCs precursors [27] (Figure 1). OPG, through RANKL binding, removes it from interaction with RANK, blocking downstream intracellular signal transduction and thus OCs genesis [28].

Intracellular signal transduction is mediated by the TNF receptor-associated factor 6 (TRAF6) that links the cell surface receptors to downstream kinase cascades, with the activation of transcription factors, such as Nuclear Factor kB (NF-kB) and Activator Protein-1 (AP-1) [29], Mitogen-Activated Protein Kinase (MAPK) and an anti-apoptotic program via c-Src-Akt/Protein Kinase B (PKB) [26]. Therefore, TRAF6 is a crucial factor for OCs formation and activation [30], as shown in a study on mice with inactivating mutation of TRAF6 (TRAF6-/-) with severe osteopetrosis [31].

The recruitment of the family of MAPK leads to the nuclear translocation of c-Fos and c-Jun transcription factors [32], while NF-kB acts as a costimulatory signal for the activation of c-Fos. The importance of c-Fos activation is proven by in vivo studies showing how homozygous c-Fos-/- mutant mice exhibit growth-retarded, severe osteopetrosis and tooth eruption [33].

The transcription factor of c-Fos, together with Nuclear Factor F of Cytoplasmic Activated T cells (NFATc1), triggers the transcription of the genetic program of the OCs genesis. For its activation, NFATc1 requires the intracellular release of calcium ions by phospholipase Cγ2 (PLCγ2). In in-vitro differentiation assays of OCs using embryonic stem cells, cells deficient in NFATc1, showed a defect in the genesis of OCs [34].

Recently, a new RANKL receptor, the leucine-rich G-protein-coupled receptor 4 (LGR4), was discovered that negatively regulates OC differentiation [35]; this receptor enhances bone formation by increasing OB maturation and mineralization, and activates the Wnt/β-catenin pathway [36].

Several studies have demonstrated increased inflammatory activity and bone resorption in CF patients, with evidence of elevated serum levels of inflammatory markers, such as TNF-α, Interleukin-1β (IL-1β) and IL-6, and bone resorption markers, such as RANKL [37,38,39]. Additionally IL-1β and IL-6 play important roles in the pathogenesis by inducing an overproduction of RANKL in CF [37]. In addition, an immunohistochemical analysis of bone samples showed increased OC activity, demonstrated by the presence of a high amount of IL-1, IL-6 and TNF-α [38].

Mabilleau et al. for the first time demonstrated the importance of the (RANKL) pathway in the pathogenesis and treatment of the CF [40]. In the bone, the clinical presentation of serum overexpression of RANKL is an increased activity of OCs, resulting in bone loss; this is also evident in other degenerative diseases, such as osteoporosis, rheumatoid and psoriatic arthritis, in addition to CF [41,42,43]. RANKL is also closely related to bone metastases from tumors such as prostate and breast cancer or multiple myeloma [44]. Breast and prostate cancer cells have been shown not only to express RANK but also to upregulate RANKL expression by OBs and bone marrow stromal cells [45].

A further area of research focused on the allelic locus in CF patients. Burakowska et al. demonstrated that some allelic loci polymorphisms of RANKL and OPG in diabetic patients lead to an increased likelihood of developing CF (in particular OPG 245T/G and OPG 1217C/T are more highly expressed in patients with CNO) [46]. Confirming this, Kloska et al. showed how different alleles coding for OPG and RANKL with different types of monocyte methylation have increased the serum expression in CF (the allelic variants associated with CNO are OPG 245T>G, 1181G>C and 1217C>T and RANKL 290C>T, 643C>T and 693G>C) [47].

Furthermore, an increased RANKL-OPG ratio in the blood is specific for neuropathy and could increase the risk of developing CF disease [46].

## 3. Therapeutical Implications on RANKL-RANK-OPG Signaling Pathway

The treatment of the acute phase of CF, aimed at resolving the painful symptomatology and controlling the local inflammatory response, is based on non-surgical strategies to reduce the load and edema of the affected foot.

The gold standard of conservative non-pharmacological treatment, based on no weight-bearing and immobilization of the foot and ankle, is the Total Contact Cast (TCC), a cast with padding evenly distributed over the entire limb, but with reinforcement on the tibial crest that is malleolus and around the metatarsal head [11,48,49].

In recent years, the pharmacological treatment of CF has been related to RANKL-RANK-OPG signaling and the OC genesis process, acting at different levels of the pathway [50]. Drugs with direct action on this pathway that have been studied in the literature are anti-RANKL monoclonal antibodies (Denosumab), Bisphosphonates (BP), and Calcitonin [51,52,53,54,55].

Despite the growing interest in this pathway in the pathogenesis of CF, there are few studies in the literature (search via PubMed and Web of Science) on the treatment of CF with these drugs, and these are mostly small-sample studies with a low level of evidence.

### 3.1. Denosumab

Denosumab is a human monoclonal antibody that selectively binds with high affinity to RANKL, preventing activation of its RANK receptor on the surface of OCs, resulting in the inhibition of any OC activity and the reduction of bone resorption [53,54,55]. Its pharmacokinetics are dose dependent, and the therapeutic effect is achieved with a single subcutaneous dose of 60 mg, which is the standard dose used in all studies reported in the literature on the treatment of CF [55]. Encouraging results have been reported with the use of Denosumab in patients with CF, and it has recently become the main treatment in the refractory active CNO stages of CF [56,57] (Table 1).

- Busch-Westbroek et al. conducted an observational study of 22 patients to evaluate the efficacy of Denosumab on CF. All patients were treated with no weight-bearing, weekly TCC changes, daily calcium supplementation (500 mg/colecalciferol 800 IU—international units), and subsequent radiographs every 4 weeks. A treatment group of 11 patients received a single subcutaneous dose of 60 mg of Denosumab. At 12 months, the patients in the treatment group showed a decrease in subchondral lysis, an improvement in subchondral bone resurfacing, and a decrease in soft tissue oedema, assessed on conventional radiographs of the affected foot. The TCC time was shorter on average in the treatment group in relation to a faster decrease in 2 °C temperature between the two feet [58].
- Shofler et al. enrolled seven patients in the acute phase of CF and followed them for one year (with biweekly visits). Patients received a single 60 mg subcutaneous dose of Denosumab and treatment with no weight-bearing and TCC. Efficacy was assessed as the subjects’ exit from the acute phase, defined by normalization of skin temperature by 2 °C relative to the contralateral foot. Patients responded to treatment at an average of 52 days after injection [59].
- Carvès et al. studied seven patients in the refractory CN stage that were treated with a single subcutaneous dose of 60 mg of denosumab (in case of concomitant osteoporosis, the injection was repeated after 6 months). The follow-up evaluation included clinical, biological examinations and imaging (radiographs and/or glucose analogue (18)F-fluorodeoxyglucose PET-CT). An imaging follow-up was available for five patients and, in four of them, structural damage remained stable on X-ray. PET-CT at baseline was available for all patients, six of whom had increased bone uptake. At the end of treatment, a significant decrease in contrast medium uptake was observed at the joints of the feet. Therefore, denosumab showed a metabolic/anti-inflammatory effect, as measured by 18FDG PET-CT, without adverse events or hypocalcaemia [57].

### 3.2. Bisphosphonates (BPs)

BPs owe their name to the presence of two phosphonate groups in their chemical structure and constitute a class of drugs with anti-OC action widely used to prevent bone mineral density loss. In recent years, several authors have focused their studies on BPs in CF treatment.

BPs inhibit the action of OCs acting on the enzyme of the cholesterol biosynthesis pathway (farnesyldiphosphate synthase, responsible of geranyl-geranylation, bundling of lipids to regulatory proteins), inhibit their proliferation and shorten their half-life [60]. Another, more recently hypothesized, mechanism of action is that BPs with imidazole groups may be directly involved in the down-regulation of c-Jun and Akt-PKB and the consequent inhibition of c-Fos and NFATc1 expression [61]. Several studies have tested the efficacy of different BPs, initially focusing on pamidronate, later shifting to alendronate and zolendronate (Table 2).

In recent years, the use of BPs has been stigmatized and limited in relation to the clinical corollary of adverse effects reported in the literature, such as the deterioration of renal function, which is particularly important in patients with diabetic neuropathy, and osteonecrosis of the jaw [62]. BPs have shown moderate efficacy in regard to the reduction of bone turnover and the reduction of skin temperature; furthermore, there are as yet no studies confirming efficacy with respect to the reduction of skin deformities and ulcerations [63,64].

- In 2001, a randomized, double-blind, controlled trial of 39 diabetic patients by Jude et al. compared treatment with a single dose infusion of pamidronate 90 mg versus placebo; the treatment group showed a reduction in bone turnover (measured as a reduction in bone-specific alkaline phosphatase and dehydroxypyridinoline) and, most importantly, a reduction in symptoms related to diabetic neuropathy [65].
- In a retrospective study, Pakarinen et al. analyzed the medical records and radiographs of 36 feet (32 patients) with acute phase CF. Eighteen received treatment with pamidronate (30–60 mg i.v. once a week for 6 weeks) and a plaster cast without weight-bearing. No significant differences were found in the two groups at the last follow-up [66].
- In 2005, Pitocco et al. conducted a study of 20 patients in the acute phase of CNO. All patients received a TCC boot for the first 2 months and a pneumatic walker for the other 4 months, then 11 patients were treated with 70 mg alendronate by mouth once a week (test group), and nine control subjects were followed for 6 months. At six months, the authors reported the significant reduction of bone reabsorption markers with increased foot bone density compared with the control group (more evident in the distal phalanxes than in the midfoot) [67].
- In 2007, in a prospective study of seven patients, Moreno et al. found a rapid resolution of clinical symptoms, with a marked reduction in all markers of bone remodeling and radiological healing at final follow-up following treatment with three doses of pamidronate at 0, 2 and 4 months [68].
- With the same protocol (three pamidronate 90 mg administrations two months apart), Naqvi et al. showed satisfactory results in terms of resolution of symptoms and ability to walk with load in three cases of CF (two in the subacute phase and 1 in the acute phase) [69].
- In 2011, a randomized double-blind controlled trial (RCT) by Pakarinen et al. compared zolendronate (4 mg i.v. in three administrations over 3 months) and foot immobilization vs. placebo, in a population of 35 patients. The use of zolendronate has not shown efficacy in the clinical resolution of CNO; rather, patients in the treatment group required a greater number of immobilization days [70].
- A three-arm double-blind RCT between methylprednisone, zolendronate and placebo showed how the use of cortisone prolonged the time to remission compared to zolendronate and placebo. Inflammatory markers decreased in the three groups, but bone resorption increased in patients treated with methylprednisone, resulting in overall bone loss. Therefore, no benefit was observed in treatment with zolendronate for CF remission [71].

**Table 2:** Studies on the treatment of CF with Bisphosphonates.

Author, Year	Partecipants	Treatment	Results
Jude et al., 2001 [65]	39	Pamidronate 90 mg (single dose) vs. Placebo	Reduction of symptoms related to diabetic neuropathy in treatment group
Pakarinen et al., 2002 [66]	32	Pamidronate 30–60 mg (i.v., once a week for 6 weeks)	No differences between the two groups
Pitocco et al., 2005 [67]	20	- All patients received a TCC boot for the first 2 months and pneumatic walker for the other 4 months - 11 patients treated with Alendronate 70 mg orally once a week (test group) - 9 control subjects followed for 6 months	At 6 months, significant reduction of bone reabsorption markers with increased foot bone density compared with the control group (more evident in the distal phalanxes than in the midfoot)
Moreno et al., 2007 [68]	7	Pamidronate 90 mg (i.v., 3 doses at 2 months-interval)	Rapid resolution of symptoms, marked reduction of bone remodeling and radiological healing at final follow-up
Naqvi et al., 2008 [69]	3	Pamidronate 90 mg (i.v., doses at 2 months-interval)	- Improvement in swelling, pain, erythema and warmth - Patients able to bear weight on their foot and no longer required the use of a walking aid
Pakarinen et al., 2011 [70]	35	Zolendronate 4 mg (i.v., 3 doses in 3 months) and foot immobilization vs. placebo	- No significative difference in the two groups - The treatment group required more days of immobilization.
Das et al., 2019 [71]	36	Methylprednisone vs. zolendronate vs. placebo	- No benefit was observed in treatment with zoledronate for remission of CN - Methylprednisone group had a worse prognosis

(mg: milligrams; i.v.: intravenous; TCC: Total Contact Cast).

### 3.3. Calcitonin

Calcitonin is a polypeptide secreted by the parafollicular C cells of the thyroid gland and acts directly on OCs by inhibiting their bone resorption activity [72]. It is used less in clinical practice than BP and denosumab due to the means of administration (mainly intranasal) [11,49].

- In a 2006 randomized double-blind study (Table 3), a daily dose of 200 IU calcitonin nasal spray and oral calcium supplementation (treatment group) was compared with oral calcium supplementation alone (control group). Calcitonin treatment showed a good effect on bone turnover at 3 months, with a significant reduction in alkaline phosphatase, but not statistically significant results on diabetic neuropathy control. Foot skin temperature was reduced in both groups, with no significant differences between the two groups [73].

## 4. Conclusions

Knowledge about the pathogenesis of CF has improved considerably in recent years. Data collected in the literature confirm the central role of the RANKL-RANK-OPG signaling pathway and the importance of this target in the treatment of bone loss and inflammatory signs, therefore research has increased on treatments aimed at interrupting this pathway. Treatment with denosumab has shown encouraging data, although in a few studies and on small sample sizes. In contrast, treatment with BPs has been re-evaluated in recent years in relation to the high possibility of side effects. Calcitonin has shown little efficacy on CNO and is little used for the mode of administration.

## Figures and Tables

**Table 1:** Studies on the treatment of CF with Denosumab.

Author, Year	Participants	Treatment	Results
Busch-Westbroek et al., 2017 [58]	22	Denosumab 60 mg (single subcutaneous dose) and TCC protocol	Fracture resolution and shorter TCC treatment time
Shofler et al., 2021 [59]	7	Denosumab 60 mg (single subcutaneous dose) in the CF acute phase	Exit from the acute phase in an average of 52.00 ± 17.89 days after injection
Carvés et al., 2021 [57]	7	Denosumab 60 mg (single subcutaneous dose) in refractory stage	- All patients clinically improved - 5 patients showed stability of structural damage (radiography) - 4 patients with significant decrease of metabolic activity (PET-CT) - No adverse event or hypocalcemia was observed.

(mg: milligrams; TCC: Total Contact Cast).

**Table 3:** Studies on the treatment of CF with calcitonin.

Author, Year	Partecipants	Treatment	Results
Bem et al., 2006 [73]	32	Calcitonin spray 200 IU + calcium vs. calcium alone	- Reduced bone turnover at 3 months - No significative difference between the two groups in foot-temperature reduction

(IU: International Units).
